# Executive Functions Assessment Based on Wireless EEG and 3D Gait Analysis During Dual-Task: A Feasibility Study

**DOI:** 10.1109/JTEHM.2024.3357287

**Published:** 2024-01-22

**Authors:** Pasquale Arpaia, Renato Cuocolo, Allegra Fullin, Ludovica Gargiulo, Francesca Mancino, Nicola Moccaldi, Ersilia Vallefuoco, Paolo De Blasiis

**Affiliations:** Department of Electrical Engineering and Information TechnologiesUniversity of Naples Federico II 9307 80138 Naples Italy; Department of Medicine, Surgery, and DentistryScuola Medica SalernitanaUniversity of Salerno 19028 84084 Salerno Italy; Department of Mental and Physical Health and Preventive MedicineSection of Human AnatomyUniversity of Campania Luigi Vanvitelli 18994 Caserta 81100 Naples Italy; Department of Advanced Biomedical SciencesUniversity of Naples Federico II 9307 80138 Naples Italy; Department of Psychology and Cognitive ScienceUniversity of Trento 19034 38122 Rovereto Italy

**Keywords:** Gait analysis, working memory, inhibition, EEG, dual task

## Abstract

Executive functions (EFs) are neurocognitive processes planning and regulating daily life actions. Performance of two simultaneous tasks, requiring the same cognitive resources, lead to a cognitive fatigue. Several studies investigated cognitive-motor task and the interference during walking, highlighting an increasing risk of falls especially in elderly and people with neurological diseases. A few studies instrumentally explored relationship between activation-no-activation of two EFs (working memory and inhibition) and spatial-temporal gait parameters. Aim of our study was to detect activation of inhibition and working memory during progressive difficulty levels of cognitive tasks and spontaneous walking using, respectively, wireless electroencephalography (EEG) and 3D-gait analysis. Thirteen healthy subjects were recruited. Two cognitive tasks were performed, activating inhibition (Go-NoGo) and working memory (N-back). EEG features (absolute and relative power in different bands) and kinematic parameters (7 spatial-temporal ones and Gait Variable Score for 9 range of motion of lower limbs) were analyzed. A significant decrease of stride length and an increase of external-rotation of foot progression were found during dual task with Go-NoGo. Moreover, a significant correlation was found between the relative power in the delta band at channels Fz, C4 and progressive difficulty levels of Go-NoGo (activating inhibition) during walking, whereas working memory showed no correlation. This study reinforces the hypothesis of the prevalent involvement of inhibition with respect to working memory during dual task walking and reveals specific kinematic adaptations. The foundations for EEG-based monitoring of cognitive processes involved in gait are laid. Clinical and Translational Impact Statement: Clinical and instrumental evaluation and training of executive functions (as inhibition), during cognitive-motor task, could be useful for rehabilitation treatment of gait disorder in elderly and people with neurological disease.

## I. Introduction

Executive functions (EFs) are neurocognitive processes needed to organize, plan and regulate daily life actions [1]. According to Diamond et al. [2], the basic EFs are working memory, inhibition and cognitive flexibility. The working memory is the ability to keep in mind information while performing complex tasks [3]. The inhibition allows to control thoughts, behavior, and/or emotions by overcoming a strong internal predisposition or external pull [2]. The cognitive flexibility is the ability to adapt to rapidly varying circumstances [4].

EFs represent multifaceted cognitive phenomena and, consequently, are not related to specific area of the brain. Indeed, several brain regions have shown a non-random association with executive functions [5]. In particular, EFs are associated with parietal lobes, limbic areas, subcortical areas, frontal lobes, prefrontal lobe, prefrontal cortex, and cingulate cortex [6]. Dysfunctions of these neuronal circuits were related to the inability to manage daily activities in the elderly and in patients with neurological disorders [7], [8]. Therefore, the monitoring and the training of the impaired EFs could be useful for early diagnosis and treatment of neurological diseases, respectively.

**FIGURE 1.**
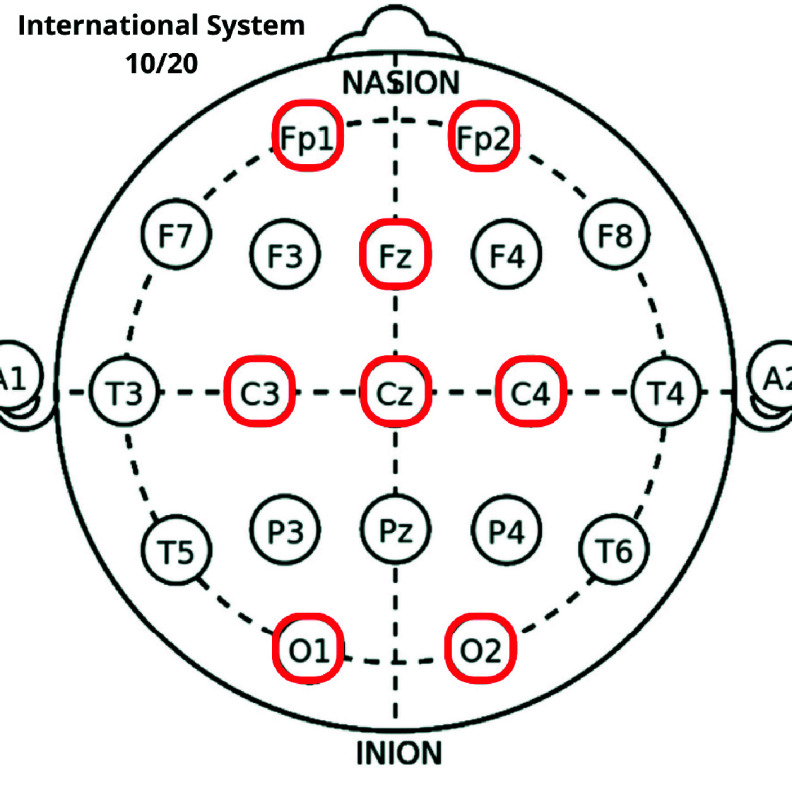
International System 10/20. The channels considered by the wireless EEG device (ab medica Helmate) are circled in red.

Recently, approaches are emerging to improve the effectiveness of rehabilitation by discriminating the specific targeted EF [9]. Several bio-markers are proposed in the literature for EFs detection. Among them, electroencephalographic (EEG)-based methods are becoming increasingly important [10], [11], [12] thanks to their high temporal resolution and good real-time performance [13]. The most discussed EEG features in the literature for the EFs analysis are the power spectral density (PSD), in different bands [14], [15], [16], at the following channels: Fz, Cz, Pz [17], [18], [19], [20], FP2 and FP1 [20], [21], [22], C4 and C3 [22], O1 and O2 [20], according to International System 10/20 (Fig. 1) [23].

The mental fatigue is a gradual and cumulative process associated with a decline in the mental efficiency due to excessive mental and/or physical activities, an impaired mental performance, or feelings of disinclination for any effort [24], [25], [26], [27], [28]. EEG-based methods are widely used in the investigation of mental fatigue being sensitive to EFs [29], [30], [31]. In particular, EEG spectral bands variations have been extensively studied [32], [33] at Fz, Cz, Pz, Poz [13], [19], [34], [35], C3 [13] channels.

The ability to carry out cognitive tasks while simultaneously walking is one of the most essential skill for daily-life activities [36]. From last years, gait has been no longer considered as an automatic activity but as an activity involvig EFs. Indeed, many studies revealed an increase in cognitive-motor interference due to a greater complexity of the cognitive task [37], [38], [39], [40].

According to the attentional capacity theory, people have limited cognitive capacity [41]. Consequently, the performance of two simultaneous tasks, requiring the same cognitive resources, leads to a decrease in efficiency on one or both (dual task effect) [42], [43]. Moreover, the increase in the complexity of one or both tasks may led to a cognitive fatigue or exhaustion of cognitive resources [44], [45]. As a result, the increase in cognitive-motor interference may cause increased risk of falls and loss mobility, especially in elderly and people with neurological diseases [46]. For these reasons, rehabilitation of motor skills can benefit from EFs reinforcement. In particular, therapies can be more effective by focusing on the specific impaired EF.

Interaction between EFs and motor tasks is widely investigated [2], [47], [48], [49], [50], especially by means of dual task [51]. Fingers motor tasks [37], driving simulated tasks [41], [43] and different levels of walking were explored [48], [49], [50], [52], [53], [54], [55]. The most analysed gait features are spatial-temporal parameters, such as velocity, step and stride lengths [48], [50], as well as EMG signals [52] for the identification of muscle time activation.

The EEG feature most investigated for cognitive activation and the EFs detection during walking is the PSD in several bands (alpha, beta, gamma, delta and theta) [52], [53], [55], [56], [57], [58]. To the authors’ knowledge, there are very few contributions investigating the selective activation of EFs in a dual task context by analyzing the EEG signal, increasing the difficulty of the task. In particular, in [52], [53], [55], and [58], no concurrent cognitive task is considered. In [56] and [57], authors considered a variation in the difficulty of the path during walking and not in the cognitive task. Moreover, some limitations emerged from those studies. As far as the EEG detection, a limit concerns high number of wet electrodes and wired EEG devices, resulting in low wearability. Regarding the gait analysis, studies were focused on forced and bound in place walking (e.g., treadmill walking) [57]. In this way, the spontaneous walking was not represented properly. Moreover, the analysis is limited to the spatial-temporal parameters by excluding the kinematic ones.

Recently, few studies aimed to understand which EFs are specifically involved during walking without considering the EEG measurements. In [59], relationships between cognitive functions and walking were explored by means of fNIRS measurements and gait speed. Both neurophysiological and motion analysis highlighted a prevalent involvement of inhibition with respect to working memory during gait. However, only the prefrontal cortex was explored and gait analysis was limited to the gait speed assessment. Moreover, only the activation-no activation of EFs were compared. Eventually, evidence of previous study [59] showed the invariance of neurophysiological features with ageing, with regard to the activation of cognitive functions in dual-tasks.

This feasibility and exploratory study aims to perform an EEG-based investigation of EFs activation, referred to progressive difficulty levels of cognitive tasks during walking for healthy young subjects, in order to laid the foundation of EEG-based monitoring of cognitive processes involved in gait. A spontaneous walking set up is achieved by means of a lightweight and wireless EEG device with few channels and dry electrodes. Despite the few electrodes, four different cortical areas are monitored (prefrontal, frontal, medial, occipital). Quantitative gait assessment is performed by a gold standard 3D motion analysis which allowed to process both spatial-temporal and kinematic data. For the selective activation of inhibition and working memory, standardised cognitive tasks are employed. Moreover, the subject is enabled to execute the cognitive task by minimizing artefacts produced by vocal or gestural responses.

## II. Methodology

In this section the EEG instrumentation and 3D motion analysis system, the experimental protocol, and the EEG and gait data processing are presented.

### A. Experimental Sample

Thirteen healthy subjects (5 females and 8 males, 24 ± 3 years) were involved in the study according to the following inclusion criteria: $18.50 < BMI < 24.99 kg/m^{2}$, lack of pain, right limb dominant, no muscle-skeletal injuries in the last 3 months, no surgical interventions in the last 6 months, no skeletal dysmorphism, no gait disorders and no cognitive impairment.

The volunteers were informed in detail about the goal of the experiment and signed the informed consent form for authorizing the inclusion in the study. All procedures were conducted in compliance with the Helsinki declaration. The Ethics Committee of Psychological Research of University of Naples Federico II approved the research.

### B. Experimental Protocol

**FIGURE 2.**
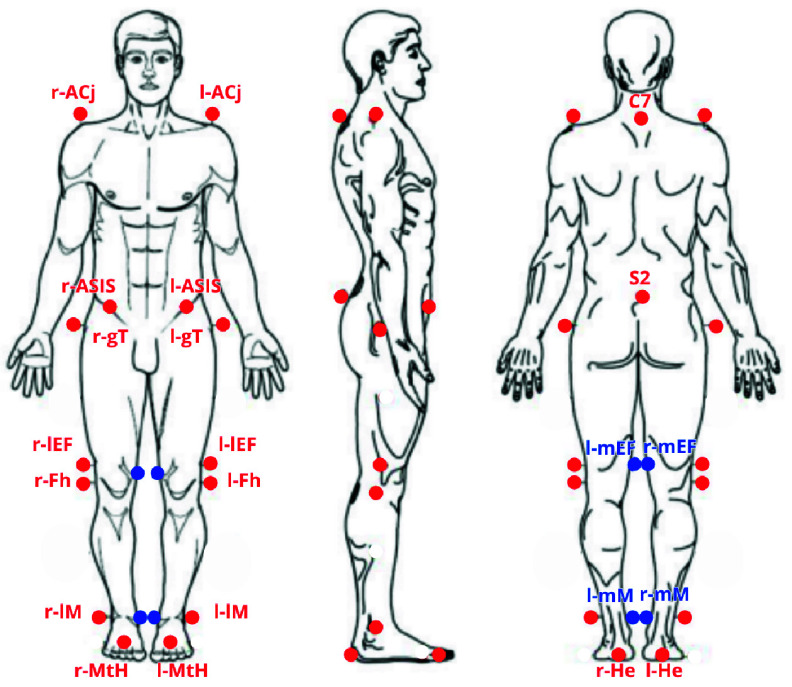
Helen Hayes MM marker set protocol. (l) left, (r) right, (S) sacrum, (C) cervical, (ACj) acromioclavicular joint, (ASIS) anterior superior iliac spine, (gT) greater trochanter, (mEF) medial and (lEF) lateral epicondyli femoris, (Fh) fibular head, (mM) medial and (lM) lateral malleoli, (MtH) metatarsal head, (He) heel.

**FIGURE 3.**
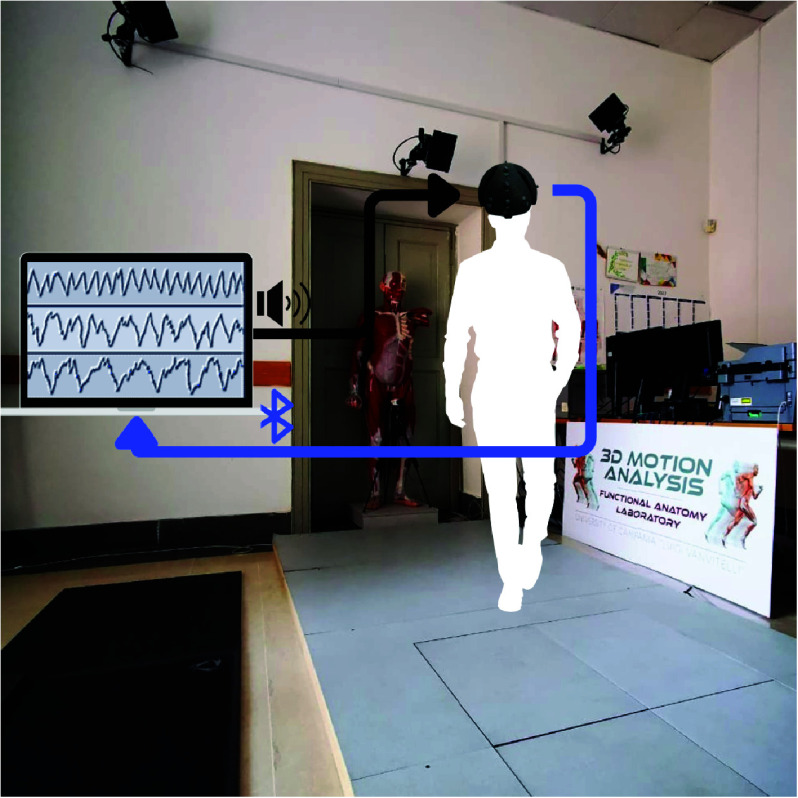
Graphical representation of dual task walking acquisition using wireless EEG (ab medica Helmate) and 3D Motion Analysis system. Acoustic stimuli of cognitive tasks are transmitted from the PC speakers to the subject being examined. A wireless receiver records EEG signals and responses to acoustic stimuli.

The experimental protocol was composed by two parts. In the first part, cognitive tasks were described to the participants, subsequently the device for EEG acquisition was set up and the subjects were required to execute the cognitive tasks by sitting on a chair. In the second part, 22 reflective markers for gait analysis were placed according to Helen Hayes MM protocol (Fig. 2) and the cognitive tasks were executed during walking (Fig. 3). The participants came into a silent room and were asked to perform the cognitive tasks by holding a wireless controller with their right hand. The wireless controller was chosen over the voice response to minimise muscle artefacts. Cognitive tasks employed for this study were the following:

• Go-NoGo task. The task mainly activates inhibition. Subjects had to respond (by pressing the button on the controller) or inhibit a response (by not pressing the button on the controller) depending on whether a ‘go’ or ‘no-go’ stimulus (i.e., trial) was heard. 150 stimuli were heard by the subjects. The decrease in the time interval between stimuli led to an increase of the task difficulty. The task was performed at two levels of difficulty: with 2-s and 1.3-s inter-trial distance in the Go-NoGo_1 and the Go-NoGo_2, respectively. Therefore, the Go-NoGo_1 lasted 300 s and the Go-NoGo_2 lasted 195 s.
• N-Back task. The task mainly activates working memory. A sequence of 75 stimuli (e.g., letters) was presented to the subjects. For each stimulus (i.e., trial), they had to decide whether the current stimulus was identical to the one heard N trials before. The difficulty of the task varied according to the load factor N. The task was performed at two levels of difficulty: with N = 1 and N = 2 in the N-Back_1 and the N-Back_2, respectively. Therefore, the N-Back_1 and the N-Back_2 lasted approximately 113 s each.

The task stimuli were acoustically provided to guarantee a natural walking during the execution of the cognitive tasks. Acoustic stimuli for cognitive tasks are transmitted from the PC speakers to the subject being examined. A wireless controller is connected to PC and receives responses of the subject to acoustic stimuli of cognitive tasks by pressing or not the button on the controller. Moreover, the EEG wireless device is connected via Bluetooth to PC that records the signal during the acquisition (Fig. 3). Each subject was asked to perform, (i) during sitting and (ii) during walking, the following steps:

• without cognitive tasks;
• executing the Go-NoGo_1;
• executing the Go-NoGo_2;
• executing the N-Back_1;
• executing the N-Back_2;

In condition (i) the subject is sat in a comfortable chair with open eyes, while in condition (ii) the subject performs a barefoot walking back and forth on a 6 m path, following an elliptical trajectory, at a self-selected normal speed and gait analysis was executed.

### C. Hardware and Software

EEG data were collected with the wireless device “ab medica Helmate” [60], certified for clinical applications. The device is an ultralight foam helmet composed by 10 dry electrodes positioned, according to the International system 10/20, on 8 active channels: Fp1, Fp2, Fz, Cz, O1, O2, C3, and C4 (Fig. 1). AFz and Fpz are reference and ground, respectively.

Customised software was developed to provide the cognitive tasks, acquire and monitor the EEG signal [61]. Quantitative gait assessment was performed using a 3D optoelectronic system consisting of eight Smart-D cameras at frequency of 100 Hz (BTS Bioengineering, Milan, Italy), for the calculation of spatial-temporal and kinematic parameters. 3D-stereophotogrammetric analysis was conducted using Helen Hayes M.M. markers set protocol [62], including 22 markers placed on the following body landmarks (Fig. 2): spinous processes of C7 and S2, acromioclavicular joint, anterior superior iliac spine, greater trochanter, medial and lateral epicondyli femoris, fibular head, medial and lateral malleoli, II metatarsal head and heel bilaterally.

### D. EEG Data Processing

In the pre-processing phase, the fourth-order Butterworth bandpass filter [0.5 - 45] Hz was applied to the EEG data. Then, the removal of transient artefacts were performed by means of the Artifact Subspace Reconstruction (ASR) [63] with a cutoff of 15. Firstly, this method decomposes a signal into parts. Subsequently it automatically defines a threshold according to the variance distribution of the signal. Then, it discards noisy components above the threshold. Finally, it uses the remaining components to reconstruct the signal. After ASR application, the EEG traces were divided into epochs. The length of each epoch depends on the duration of the trial: in the N-Back task, each trial lasts 1.5 s at both levels of difficulty, while, in the Go-NoGo task, trials last 2 s or 1.3 s, for the first and second level of difficulty, respectively.

The EEG features extracted from the signal were the absolute [ $\mu V^{2}$] and relative power [%] computed on PSD function in the alpha ([8-13] Hz), theta ([4-8] Hz), beta ([13-30] Hz), low beta ([13-20] Hz), high beta ([20-30] Hz), gamma ([30-45] Hz) and delta ([1-4] Hz) bands for 8 active channels (112 EEG features). Then, for each EEG feature, the mean values over all the epochs were computed for the steps described in Section II-B.

Statistical analysis were performed on all the extracted EEG features in order to find their relationship with progressive difficulty levels of cognitive tasks, activating inhibition (Go-NoGo) and working memory (N-Back). Four analysis contexts were identified by combining the condition (sitting or walking) with the cognitive task (N-Back or Go-NoGo). For each analysis context, EEG features were considered in 3 progressive difficulty levels of 2 cognitive task execution: (1) no cognitive task execution, (2) first, and (3) second level of the cognitive task difficulty. The inter-subject Spearman rank correlation coefficients were computed to find a monotonic relationship between EEG features and progressive difficulty levels of cognitive tasks, activating inhibition (Go-NoGo) and working memory (N-Back). Finally, the EEG features, revealing the Spearman rank correlation coefficient equal to 1 or −1 (positive or negative monotonic correlation), were subjected to a Friedman test to determine which of these correlations were statistically significant ( ${p} < 0.05$). All statistical analysis were performed using R [64].

### E. 3D Gait Data Processing

The raw data were processed by the Smart Analyzer (BTS-Bioengineering, Milano, Italy). Seven spatial-temporal parameters were calculated: cadence [step/min], gait speed [m/s], stance, swing and double support phases percentage [%], stride length [m], step width [m]. Moreover, nine kinematic (k) parameters were computed, referred to Gait Variable Scores (GVS) for lower limbs range of movement: pelvic tilt, rotation and obliquity, hip flexion-extension, adduction-abduction and rotation, knee flexion-extension, ankle dorsiflexion and foot progression. Finally, the Gait Profile Score (GPS) was obtained by the sum of the root mean square (RMS) of differences between a patient’s data and a reference value related to a population of healthy individuals [65]. Higher GVS and GPS scores indicate larger deviations from a physiological gait.

Statistical differences between left and right sides were tested via non-parametric Wilcoxon signed-rank tests for all spatial-temporal and kinematic parameters. Mean values and Standard Deviations (SDs), across all trials of each session, were calculated for the spatial-temporal and k parameters. Inter-subject significant differences for all features were tested using Wilcoxon-Mann-Whitney test for six different comparisons (Walking vs N-Back_1, Walking vs N-Back_2, Walking vs Go-NoGo_1, Walking vs Go-NoGo_2, N-Back_1 vs N-Back_2 and Go-NoGo_1 vs Go-NoGo_2). All statistical analysis were performed using R [64].

## III. Results

### A. EEG Results

According to the Friedman test ( $\alpha = 0.05$), 27 EEG features for inhibition and 16 for working memory exhibited a statistically significant positive or negative correlation with 3 progressive difficulty levels of Go-NoGo (inhibition) and N-Back (working memory) in the sitting condition. The first ten of aforementioned significant EEG features were chosen in ascending order of p-value for each cognitive task and reported in Table 1.

**TABLE 1:** EEG Features (Relative and Absolute Power in Different Bands and Channels), Showing a Significant Monotonic Correlation With Respect to 3 Progressive Difficulty Levels of Cognitive Tasks in Sitting Condition, Were Implemented via Friedman Test in Both Conditions. Results Were Reported in Terms of p-Value, Effect Size and $\chi^{2}$. Results With High Effect Size ( $\geq0.8$) and Slightly High Effect Size ( $0.5 < eff.size < 0.8$) are in Bold. $Rel$. = “Relative Power”, $Abs$. = “Absolute Power”, $n.m.$ = “no Monotonicity”

Abs. or Rel. Band	Channel	Cognitive task (EF)	Sitting Condition			Dual task walking Condition		
			p-value	eff. size	$\chi ^{2}$	p-value	eff. size	$\chi ^{2}$
Abs. high beta	Fz	N-Back (Working memory)	0.00004	0.92	20.20	0.36800	0.08	2.00
Abs. high beta	Cz		0.00043	0.65	15.50	n.m		
Abs. high beta	C4		0.00646	0.42	10.10	0.52900	0.06	1.27
Rel. low beta	O1		0.00786	0.37	9.69	0.00230	0.47	12.20
Rel. delta	Cz		0.00786	0.37	9.69	n.m		
Rel. delta	C3		0.00917	0.36	9.38	n.m		
Abs. beta	Fz		0.01160	0.41	8.91	n.m		
Rel. beta	O2		0.01250	0.34	8.77	0.02490	0.28	7.38
Rel. delta	O2		0.01250	0.34	8.77	n.m		
Abs. beta	C4		0.01690	0.34	8.17	0.69500	0.03	0.73
Rel. delta	C3	Go-NoGo (Inhibition)	0.00001	0.93	24.20	0.00917	0.36	9.38
Rel. delta	Fz		0.00002	0.82	21.40	0.00091	0.54	14.00
Rel. delta	Cz		0.00004	0.79	20.50	0.00156	0.20	12.90
Rel. delta	C4		0.00004	0.79	20.50	0.00091	0.54	14.00
Rel. delta	O1		0.00007	0.73	19.10	0.00289	0.45	11.70
Rel. delta	O2		0.00009	0.72	18.60	0.00197	0.48	12.50
Rel. delta	Fp1		0.00018	0.66	17.20	0.01830	0.31	8.00
Rel. low beta	C3		0.00046	0.59	15.40	0.03230	0.26	6.86
Rel. low beta	Fz		0.00091	0.54	14.00	0.06270	0.21	5.54
Rel. high beta	Fp2		0.00273	0.45	11.80	0.00156	0.50	12.90

**FIGURE 4.**
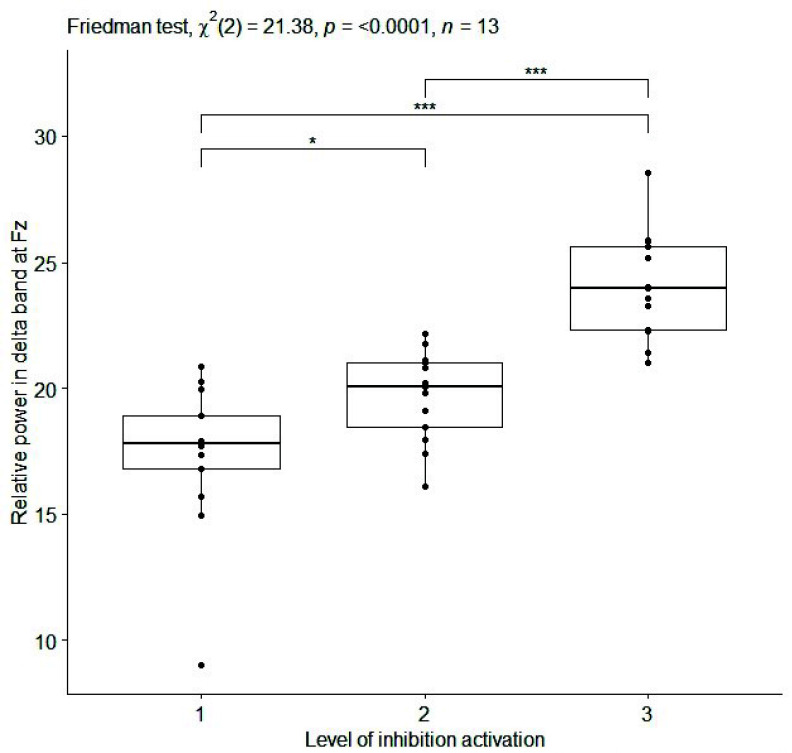
Box plot of relative power [%] in delta band at Fz (averaged on each subject) for inhibition activation at 3 progressive levels of Go-NoGo in the sitting condition. Single, double and triple asterisks denote significant difference at (p <.05), (p <.01), and (p <.001), respectively. (1) no cognitive task execution, (2) first and (3) second level of the cognitive task difficulty.

**FIGURE 5.**
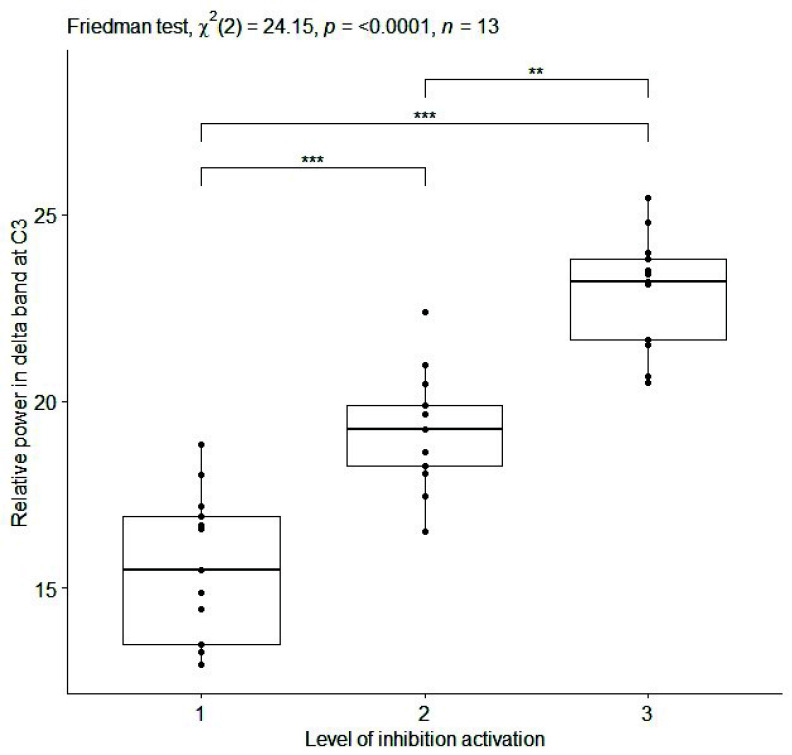
Box plot of relative power [%] in delta band at C3 (averaged on each subject) for inhibition activation at 3 progressive levels of Go-NoGo in the sitting condition. Single, double and triple asterisks denote significant difference at (p <.05), (p <.01), and (p <.001), respectively. (1) no cognitive task execution, (2) first and (3) second level of the cognitive task difficulty.

**FIGURE 6.**
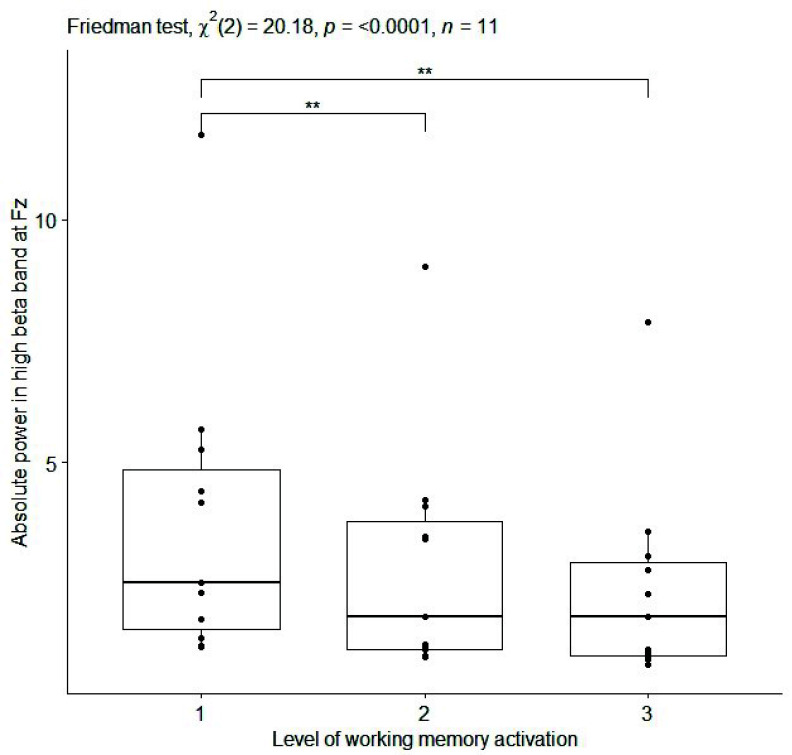
Box plot of absolute power [ $\mu V^{2}$] in high beta band at Fz (averaged on each subject) for working memory activation at 3 progressive difficulty levels of N-Back in the sitting condition. Single, double and triple asterisks denote significant difference at (p <.05), (p <.01), and (p <.001), respectively. (1) no cognitive task execution, (2) first and (3) second level of the cognitive task difficulty.

EEG features with high effect size values (i.e., $\geq0.8$, based on benchmarks suggested by Cohen [66]) were: relative power in delta band at Fz during Go-NoGo execution by sitting ( $\chi ^{2}(2)=21.40$, ${p}= 0.0002$, effect size=0.82), relative power in delta band at C3 during Go-NoGo execution by sitting ( $\chi ^{2}(2)=24.20$, ${p}= 0.0001$, effect size=0.93), and absolute power in high beta band at Fz during N-Back execution by sitting ( $\chi ^{2}(2)=20.20$, ${p}= 0.0004$, effect size=0.92) (Table 1). Boxplots of the values of the three aforementioned EEG features are reported in Fig. 4, Fig. 5, and Fig. 6 for relative delta power at Fz, C3 and Absolute delta power at Fz, respectively. Moreover, EEG features with slightly high effect size values (i.e., $0.5 < eff.size < 0.8$, as suggested by [66], [67]) in sitting condition were also reported in Table 1.

The results of the Friedman test were confirmed by a further Spearman’s rank correlation analysis conducted at the level of single subjects (Tables 2a and 2b). This analysis was restricted to the EEG features with high effect size in sitting condition (the absolute power in high beta band during N-Back and the relative power in delta band during Go-NoGo) and implemented at all channels. In particular, in Tables 2a and 2b Spearman’s rank correlation coefficients for each subject during sitting condition were reported, highlighting the negative and positive correlation at least 8/13 subjects. This analysis confirmed the negative monotonic correlation between absolute power in high beta band at Fz and 3 progressive difficulty levels of N-Back (Table 2a) and the positive monotonic correlation between the relative power in delta band at Fz, C3 and 3 progressive difficulty levels of Go-NoGo (Table 2b).

**TABLE 2:** Spearman’s Rank Correlation Coefficient Between (a) Absolute Power in High Beta Band and 3 Progressive Levels of N-Back (Working Memory) Execution in Sitting Condition, (b) Relative Power in Delta Band and 3 Progressive Levels of Go-NoGo (Inhibition) Execution in Sitting Condition, and (c) Relative Power in Delta Band and 3 Progressive Levels of Go-NoGo (Inhibition) Execution in Dual Task Walking Condition. High and Slightly High Effect Size are in Bold (“−1” and “1” Represent Negative and Positive Correlation of Ranks)

Subject ID	Coefficient of Correlation at Channels							
	$o1$	$c3$	$fp1$	$cz$	$fz$	$fp2$	$c4$	$o2$
01	−1	−1	1	−1	−1	0.5	−1	−1
02	−1	−0.5	1	−1	−1	0.5	−1	−0.5
03	−1	−1	0.5	−1	−1	−1	−1	−1
04	−1	0.5	−0.5	0.5	0.5	0.5	0.5	0.5
05	−1	1	1	0.5	−1	0.5	0.5	−0.5
06	−0.5	−0.5	−1	−1	−0.5	−0.5	−0.5	1
07	−0.5	0.5	0.5	−0.5	−1	0.5	−0.5	1
08	1	−0.5	−1	−1	−1	−0.5	−0.5	0.5
09	1	−1	−1	−1	−1	−1	−1	1
10	−1	−0.5	−1	−1	−1	−0.5	−0.5	−0.5
11	0.5	−1	1	−0.5	−1	−1	−1	1
12	−0.5	−1	−0.5	−1	−1	0.5	−1	−1
13	1	0.5	1	0.5	0.5	0.5	−0.5	0.5
(a)								

Subject ID	Coefficient of Correlation at Channels							
	$o1$	$c3$	$fp1$	$cz$	$fz$	$fp2$	$c4$	$o2$
01	1	1	−0.5	0.5	0.5	0.5	1	1
02	1	1	0.5	1	1	0.5	1	1
03	1	1	1	1	1	0.5	1	1
04	1	1	1	1	1	0.5	1	1
05	1	1	1	1	1	1	1	1
06	1	1	1	1	1	1	0.5	0.5
07	0.5	0.5	1	0.5	0.5	1	0.5	0.5
08	0.5	1	1	1	1	0.5	1	1
09	1	1	1	1	1	0.5	1	0.5
10	1	1	0.5	1	1	0.5	1	1
11	1	1	1	1	1	0.5	1	1
12	0.5	1	0.5	0.5	0.5	0.5	1	1
13	1	1	1	1	1	1	1	1
(b)	

Subject ID	Coefficient of Correlation at Channels							
	$o1$	$c3$	$fp1$	$cz$	$fz$	$fp2$	$c4$	$o2$
01	1	−0.5	−0.5	−0.5	0.5	1	1	1
02	1	1	1	0.5	1	1	1	−0.5
03	0.5	1	1	1	1	0.5	0.5	1
04	−0.5	1	1	1	1	1	1	1
05	−0.5	0.5	0.5	0.5	0.5	0.5	0.5	−0.5
06	0.5	0.5	1	1	1	1	1	1
07	1	1	1	1	1	1	0.5	1
08	0.5	1	0.5	1	0.5	1	−0.5	0.5
09	1	1	1	1	1	1	1	1
10	1	−0.5	−0.5	1	0.5	1	1	0.5
11	0.5	−0.5	−0.5	−0.5	−0.5	0.5	0.5	1
12	1	1	1	1	1	0.5	1	1
13	1	0.5	0.5	1	1	1	1	1
(c)								

**FIGURE 7.**
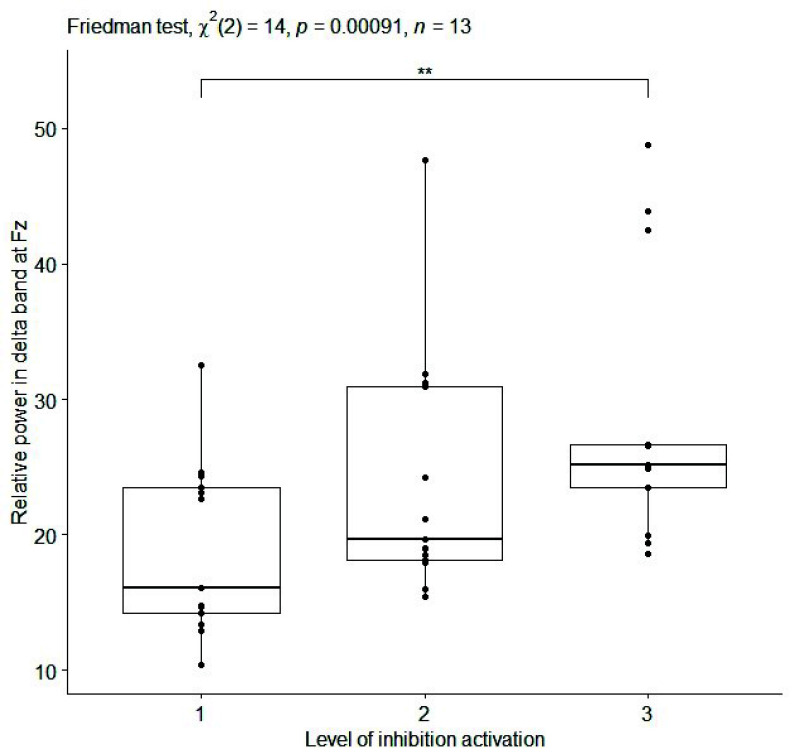
Box plot of relative power [%] in delta band at Fz (averaged on each subject) for inhibition activation at 3 progressive difficulty levels of Go-NoGo in the walking condition. Single, double and triple asterisks denote significant difference at (p <.05), (p <.01), and (p <.001), respectively. (1) no cognitive task execution, (2) first and (3) second level of the cognitive task difficulty.

**FIGURE 8.**
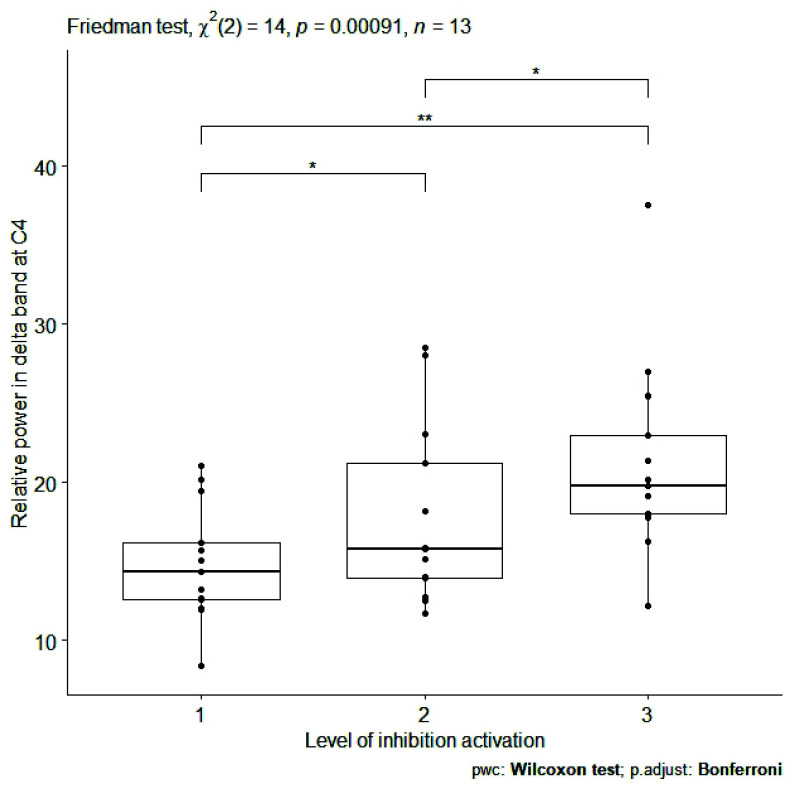
Box plot of relative power [%] in delta band at C4 (averaged on each subject) for inhibition activation at 3 progressive difficulty levels of Go-NoGo in the walking condition. Single, double and triple asterisks denote significant difference at (p <.05), (p <.01), and (p <.001), respectively. (1) no cognitive task execution, (2) first and (3) second level of the cognitive task difficulty.

The high effect size found in sitting condition was not confirmed for the same EEG features during dual task walking. Only a slightly high effect size emerged during Go-NoGo execution in dual task walking for the relative power in delta band at Fz ( $\chi ^{2}(2)=14.00$, ${p}= 0.00091$, effect size = 0.54) and C4 ( $\chi ^{2}(2)=14.00$, ${p}=0.00091$, effect size = 0.54). For these EEG features, the boxplots is reported in Fig. 7 and Fig. 8. In Table 2c Spearman’s rank correlation coefficients are showed for each subject during dual task walking for relative power in delta band at all channels. This analysis confirmed a positive monotonic correlation between the relative power in delta band at Fz and C4 and 3 progressive difficulty levels of Go-NoGo during walking.

Eventually, in the comparison of the baseline between sitting and dual task walking conditions, a monotonic increasing trend of power values was found in the transition from sitting to walking. This trend was found in all channels and bands, and could be probably due to the noise affecting EEG signals, especially during ambulation.

### B. Gait Analysis Results

**FIGURE 9.**
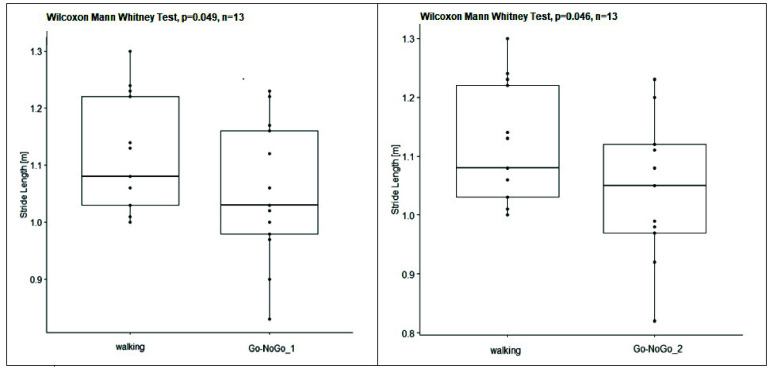
Box plots of Stride Length [m] inter-subjects distribution between (1) walking without cognitive task execution and (2) dual task walking with Go-NoGo_1 (on left) and (3) with Go-NoGo_2 (on right), respectively.

**FIGURE 10.**
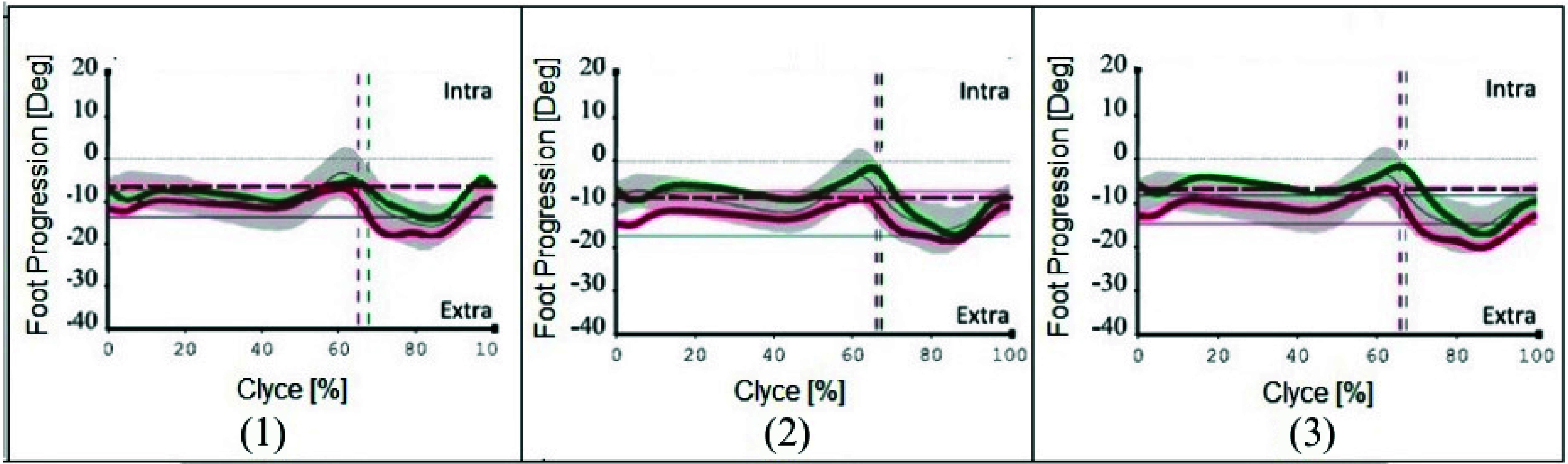
Kinematic curves of Foot Progression parameter in gait cycle, during walking without cognitive task execution (1), dual task walking with Go-NoGo_1 (2), and with Go-NoGo_2 (3). The right side and left side of the body are reported in green and red, respectively; horizontal dashed red line indicates peak of left foot progression trend; horizontal grey line is the threshold for Foot Progression parameter; horizontal red lines indicate mean values of Foot Progression for left sides; vertical dashed green and red lines delimited stance and swing phases, respectively, for left and right trends of foot progression. Deg = “Degree”, Intra = “Intra-rotation”, Extra = “External-rotation”.

Inter-side analysis showed no significant differences between left and right sides, therefore, only left gait cycles were considered for subsequent statistical analyses. Inter-subject significant differences (p-value<0.05) for spatial- temporal and k parameters (GPS and GVS) between walking without cognitive task execution and different levels of dual task walking were reported respectively in Tables 3 and 4. Table 3 showed a significant lower stride length during dual task, in particular during the execution of dual task with Go-NoGo_1 (p-value = 0.049) and Go-NoGo_2 (p-value = 0.046), with respect to walking without cognitive task execution; this finding was graphically reported via boxplots in Fig. 9. Moreover, Table 4 showed a significant lower GVS of Foot Progression during dual task walking with Go-NoGo_1 (p-value = 0.045) and Go-NoGo_2 (p-value = 0.048) with respect to walking without cognitive task execution (as showed in Fig. 10), and during dual task walking with N-Back_2 with respect to walking without cognitive task execution (p-value = 0.036). In particular, graphical representation of foot progression parameter during gait cycle was reported in Fig. 10 and highlighted a trend of foot external-rotation during cognitive-motor task with Go-NoGo.

**TABLE 3:** Results of Wilcoxon Mann Whitney Test for Spatial-Temporal Parameters Among Different Experimental Conditions. Significant Differences (p-Value < 0.05) are Highlighted in Bold

Experimental Conditions	Cycle Duration [s]	Cadence [step/min]	Gait Speed [m/s]	Stance Phase [%]	Swing Phase [%]	Double Support Phase [%]	Stride Length [m]	Step Width [m]
Walking vs. Go-NoGo_1	0.398	0.408	0.174	0.331	0.198	0.146	0.049	0.368
Walking vs. Go-NoGo_2	0.479	0.439	0.193	0.500	0.092	0.255	0.046	0.258
Walking vs. N-Back_1	0.418	0.439	0.224	0.500	0.244	0.164	0.145	0.183
Walking vs. N-Back_2	0.439	0.428	0.232	0.269	0.260	0.079	0.078	0.219
Go-NoGo_1 vs. Go-NoGo_2	0.428	0.400	0.489	0.306	0.132	0.324	0.459	0.408
N-Back_1 vs. N-Back_2	0.489	0.500	0.462	0.306	0.500	0.286	0.369	0.489

**TABLE 4:** Results of Wilcoxon Mann Whitney Test for Kinematic Parameters Among Different Experimental Conditions. Significant Differences (p-Value < 0.05) are Highlighted in Bold. GPS = “Gait Profile Score”, GVS = “Gait Variable Scores”

Experimental Conditions	GPS	GVS								
		Pelvic Obliquity	Pelvic Tilt	Pelvic Rotation	Hip Abb- Abduction	Hip Felx- Extension	Hip Rotation	Knee Flex- Extension	Ankle Dorsi- Plantiflexion	Foot Progression
Walking vs. Go-NoGo_1	0.286	0.259	0.500	0.479	0.331	0.388	0.510	0.269	0.220	0.045
Walking vs. Go-NoGo_2	0.177	0.349	0.459	0.295	0.286	0.295	0.418	0.295	0.164	0.048
Walking vs. N-Back_1	0.205	0.428	0.469	0.429	0.459	0.359	0.331	0.438	0.177	0.119
Walking vs. N-Back_2	0.369	0.408	0.469	0.489	0.408	0.340	0.379	0.500	0.252	0.036
Go-NoGo_1 vs. Go-NoGo_2	0.359	0.397	0.510	0.379	0.408	0.398	0.449	0.124	0.340	0.152
N-Back_1 vs. N-Back_2	0.349	0.489	0.448	0.479	0.459	0.379	0.510	0.388	0.379	0.236

### C. Scores of Cognitive Tasks

Results of cognitive performance of each task are reported in terms of percentage of correct answers in Table 5. In particular, N-Back consists of 44 trials while Go-NoGo of 150, therefore the percentage values are normalized on 44 and 150, respectively. The scores in percentage of the cognitive tasks exhibited high values in different difficulty levels of cognitive tasks for all subjects. This confirmed the subjects’ adherence to the experimental protocol.

**TABLE 5:** Scores (i.e., Percentage of Correct Answers) for Each Subject at Varying of Condition and Difficulty Level of Cognitive Tasks. N-Back Consists of 44 Trials While the Go-NoGo of 150, Therefore the Percentage Values are Normalized on 44 and 150, Respectively

Task	Condition	Level	Score [%] of cognitive tasks for each subject												
			1	2	3	4	5	6	7	8	9	10	11	12	13
Go-NoGo	sitting	easy	100	100	99	100	100	98	99	98	98	99	99	97	100
		difficult	100	97	99	96	94	89	93	92	96	95	93	99	94
	walking	easy	99	100	99	100	98	100	81	94	97	98	81	100	97
		difficult	96	92	98	97	95	89	95	87	95	89	76	99	91
N-Back	sitting	easy	98	100	100	95	100	100	95	98	100	98	95	100	95
		difficult	95	89	91	95	80	80	82	64	84	95	82	95	80
	walking	easy	93	93	100	100	91	95	98	100	98	95	98	98	100
		difficult	91	89	95	93	93	93	61	80	91	93	61	98	77

## IV. Discussion

Previous studies demonstrated that the performance of two simultaneous tasks, requiring the same cognitive resources, lead to a cognitive fatigue. The interaction between cognitive and motor activities was instrumentally investigated. Only the relationship between activation-no-activation of two EFs (working memory and inhibition) and spatial-temporal gait parameters was previously analyzed.

Aim of the present study was to detect activation of EFs (inhibition and working memory) in 3 progressive difficulty levels of 2 cognitive tasks (Go-NoGo and N-Back) during dual task walking using wireless electroencephalography (EEG) and 3D-gait analysis, respectively.

As far as the EEG results is concerned, monotonic relationships were identified between several EEG features and 3 progressive difficulty levels of cognitive tasks, while the subject is sitting and during dual task walking (Tables 1 and 2). In particular, during sitting condition (i), a positive correlation was found between relative power in delta band at the most of channels and 3 progressive difficulty levels of cognitive task (Go-NoGo) activating inhibition (Tables 1 and 2 b; Figs. 4 and 5). These findings are in accordance with [68] where the increase in power in the delta band is linked to the deactivation of sensory afferents to allow the subject to concentrate on the task. Instead, a high and negative correlation was found between absolute power in high beta band at Fz and 3 progressive difficulty levels of cognitive task (N-Back) activating working memory (Tables 1 and 2a; Fig. 6). This result confirms the relevance of the frontal midline for EEG-based monitoring of working memory activity [69].

EEG features, identified in sitting condition, did not maintained a statistically significant relationships with the progressive difficulty levels of cognitive tasks during dual task walking. The only exception was for the relative power in delta band at Fz and C4 channels, that showed a significant and positive correlation with respect to 3 progressive difficulty levels of Go-NoGo, activating inhibition (Tables 1 and 2c; Figs. 7 and 8). During sitting condition (i), EFs are stimulated only by the cognitive tasks. In this way, electroencephalographic interference, due to movements of the subject, are minimized. During dual task walking (ii), there is a worsening of the signal-to-noise ratio (typically of 10 dB) due to artefacts related to electrode movement. Therefore, the identification of EEG features becomes more difficult compared to sitting condition. Probably for this reason, the relationships found between EEG features and 3 progressive difficulty levels of cognitive tasks, emerged during the sitting condition, may not be confirmed during dual task walking. However, under particular experimental conditions (i.e., dual task walking with more difficult levels of cognitive exercise), the EFs might be overstimulated and the noise due to walking might be balanced by an increase in signal power.

Statistical results of gait analysis confirmed EEG ones, showing significant lower stride length during cognitive-motor task with Go-NoGo in both levels of difficulty with respect to walking without cognitive task execution (Table 3 and Fig. 9). Significant lower stride length during dual task suggested a lower balance control and more instability in subjects during walking because of execution of Go-NoGo. Subjects reduced length of their stride and, consequently, their gait speed in order to better control their gait during dual task.

Moreover, inter-subject statistical analysis for kinematic parameters underlined a significant greater GVS for Foot progression. This result was found during cognitive-motor task with Go-NoGo in both levels of difficulty with respect to walking without cognitive task execution (Table 4), highlighting a different kinematic pattern of this parameter with respect to its typical trend. In particular, an external-rotation of feet was found in gait cycle during dual task walking with Go-NoGo with respect to walking without cognitive task execution (Fig. 10) and modification of this parameter could be related to stride length significant decrease, as previously reported in literature [70]. In these conditions, an involvement of cognitive inhibition can be supposed during ambulation, underlining that both walking and Go-NoGo task executions are requiring the same cognitive resource. Eventually, a significant greater GVS for foot progression was found even during N-Back at the highest level of difficulty (N-Back_2) with respect walking without cognitive task execution (Table 4). This may indicate an involvement of working memory during motor task execution, but only in correspondence with the highest difficulty of cognitive task.

Gait results supported EEG findings, confirming a greater involvement of inhibition during gait with respect to working memory, in agreement with previous studies [71], [72], [73], and probably revealing an interference of this executive function activation on motor task execution. This is a first step toward the EEG-based monitoring of EFs fatigue during walking.

### A. Limitations

The experimental sample was composed by only 13 healthy and young participants. In future study, a greater experimental sample will be enrolled by including also elderly and people affected by neurodegenerative disorders. Cognitive flexibility was not explored due to participant sustainability concerns regarding the experimental protocol. Moreover, EEG signal was acquired by means of an eight-channel device. Therefore, the low number of the channels may have penalized the identification of additional EEG features. Furthermore, the participants were not instructed about prioritization between motor and cognitive tasks. Therefore, some inter-participant differences in performance may have occurred. Finally, dry electrodes, although they shorten device wearing time and improve participant comfort, have greater sensitivity to motion artifacts than wet electrodes.

## V. Conclusion

In this study, activation of cognitive inhibition in progressive difficulty levels of Go-NoGo task were electroencephalographically identified during a dual task (cognitive and motor) execution. This assumption is proven by the statistically significant relationships found between 3 progressive difficulty levels of cognitive task (Go-NoGo), activating inhibition, and the relative power in delta band at Fz during dual task walking. Moreover, EEG findings are in accordance with gait analysis results, that showed a significant decrease of stride length and a significant increase in external-rotation of foot progression during dual task walking with Go-NoGo execution in both levels of difficulty.

The present study lays the foundations for EEG-based monitoring of cognitive processes involved in walking. These findings could be useful to prevent falls and to provide a personalised rehabilitation in elderly people and patients with neurological diseases.

Future studies will use EEG wireless instrumentation with wet and more electrodes, involving a larger experimental sample and including elderly people and patients affected by neurological diseases, and also the cognitive flexibility will be investigated.
